# Structures and Dynamics of Complex Guest Molecules in Confinement, Revealed by Solid-State NMR, Molecular Dynamics, and Calorimetry

**DOI:** 10.3390/molecules29071669

**Published:** 2024-04-08

**Authors:** Nadia B. Haro Mares, Sonja C. Döller, Till Wissel, Markus Hoffmann, Michael Vogel, Gerd Buntkowsky

**Affiliations:** 1 Eduard-Zintl-Institut für Anorganische und Physikalische Chemie, Technische Universität Darmstadt, Peter-Grünberg-Str. 8, D-64287 Darmstadt, Germany; haromares@chemie.tu-darmstadt.de (N.B.H.M.); sonja.doeller@tu-darmstadt.de (S.C.D.); wissel@chemie.tu-darmstadt.de (T.W.); 2 Department of Chemistry and Biochemistry, State University of New York at Brockport, Brockport, NY 14420, USA; 3 Institute for Condensed Matter Physics, Technische Universität Darmstadt, Hochschulstr. 6, D-64289 Darmstadt, Germany

**Keywords:** confinement, NMR, molecular dynamics, mesoporous silica

## Abstract

This review gives an overview of current trends in the investigation of confined molecules such as water, small and higher alcohols, carbonic acids, ethylene glycol, and non-ionic surfactants, such as polyethylene glycol or Triton-X, as guest molecules in neat and functionalized mesoporous silica materials employing solid-state NMR spectroscopy, supported by calorimetry and molecular dynamics simulations. The combination of steric interactions, hydrogen bonds, and hydrophobic and hydrophilic interactions results in a fascinating phase behavior in the confinement. Combining solid-state NMR and relaxometry, DNP hyperpolarization, molecular dynamics simulations, and general physicochemical techniques, it is possible to monitor these confined molecules and gain deep insights into this phase behavior and the underlying molecular arrangements. In many cases, the competition between hydrogen bonding and electrostatic interactions between polar and non-polar moieties of the guests and the host leads to the formation of ordered structures, despite the cramped surroundings inside the pores.

## 1. Introduction

Ordered periodical mesoporous silica (PMS) [1,2], like MCM-41 (Mobil Composition of Matter No. 41) [3] and SBA-15 (Santa Barbara Amorphous) [4,5] and their many derivates, exhibit characteristic narrow pore-diameter distributions and large specific surface areas. Their high chemical stability makes them easy to handle under ambient conditions. Their reactive surface silanol groups (Si-OH) provide an easy pathway to chemical functionalization and tailored surface design, e.g., by post-synthetic grafting of functional groups such as amino, amide, carboxyl, phosphate [6,7], or by co-condensation with molecules containing such groups [8,9]. They have a high application potential in many technical processes, such as heterogeneous catalysis, separation technology, encapsulation of molecules, drug delivery, or selective adsorption [10,11,12]. Moreover, they provide an ideal model environment investigating the physicochemical properties of fluid guest molecules confined in a porous environment, showing strong competition with solid–liquid and liquid–liquid interactions or in biomineralization [13].

Revealing these properties necessitates the combination of several analytical and computational techniques, such as X-ray (XRD) and neutron diffraction techniques for the investigation of crystallinity and long-range order [14,15,16,17], small angle scattering (SAXS and SANS) for the characterization of the pore geometry and pore ordering, differential scanning calorimetry (DSC) [18] for the study of phase or glass transition processes in confinement, gas adsorption (BET, BJH) for the characterization of the pore ordering, specific surface areas and pore diameters [19,20], solid-state NMR (SSNMR), and NMR diffusometry [21,22,23,24,25], possibly supported by Dynamic Nuclear Polarization (DNP) [26,27,28,29,30,31,32,33], to boost the NMR sensitivity or chemical shift calculations to help in the interpretation [34,35], or the study of the local ordering and dynamics on the molecular level and molecular dynamics (MD) simulations for the modelling of the dynamics and structures of the confined guests in the host material.

Hydrogen-bonded liquids in nanoscale confinements are a highly topical field of research [36]. In view of their enormous relevance to science and application, particularly intensive research efforts ascertained the properties of neat water and aqueous solutions under such circumstances [37,38]. Also, the properties of confined alcohol molecules in dependence on the size and chemistry of the confining framework were often a research focus [36,39]. In such investigations, phase behaviors, structures, and dynamics of hydrogen-bonded liquids were addressed, applying a wide range of experimental and computational methods [39]. For studies of dynamical aspects, broadband dielectric spectroscopy [40], quasielastic neutron scattering [41], NMR spectroscopy and diffusometry [42], and MD simulations [43,44] were very suitable methods. Various comprehensive review articles summarized these research efforts [36,37,38,39,40,41,43,44].

Here, we review NMR approaches to the structural and dynamical properties of hydrogen-bonded liquids in porous frameworks. In doing so, we build upon several review articles covering this topic [45,46,47,48,49]. Using isotope selective approaches in NMR spectroscopy, both rotational motion and translational diffusion in confinement were successfully determined. To characterize the reorientation of confined molecules, ^2^H NMR studies of deuterated compounds proved to be a powerful tool [50,51,52]. In particular, when combining ^2^H spin-lattice relaxation (SLR) analysis, including ^2^H field-cycling relaxometry [53,54], with ^2^H stimulated-echo experiments (STE), it was possible to follow MD over a broad range of correlations times [55,56,57], *τ* ≈ 10^−11^–10^0^ s. Furthermore, NMR experiments in magnetic field gradients enabled measurements of self-diffusion coefficients [23]. In these approaches, it was advantageous to use a static field gradient (SFG) rather than a pulsed one [58,59]. The SFG method enabled an application of stronger gradients and, in this way, an observation of diffusion on smaller-length scales down to roughly 100 nm. By exploiting these capabilities, it was possible to ensure that diffusion inside a particular framework is probed, e.g., inside a given pore, whereas distorting effects from an escape of the confinement can be neglected, e.g., fast displacements in empty space between mesoporous silica particles.

While these NMR approaches provide a quantitative evaluation of the molecular motions present, their qualitative interpretation on the molecular level can be challenging. To overcome this challenge, MD simulations provide a powerful theoretical approach to gain such molecular level insights. In MD simulations, an ensemble of molecules is allowed to evolve in time to obtain essentially a movie that reveals the present dynamic processes, intermolecular interactions, and the resulting structural patterns. The potentials of all bonding and non-bonding interactions between all present atoms must be defined at each time increment in order to obtain a new set of velocities at which each atom moves during the next simulation step. In ab-initio MD (AIMD) simulations, the potentials are re-calculated ab-initio at each simulation time step [60]. The AIMD method is computationally very demanding, limiting its use to smaller-sized systems. For that reason, more commonly used are MD simulations that employ classical potential functions during the simulation. Bonding interactions typically consist of harmonic oscillator functions to describe chemical bonds and bond-angle vibrations and sinusoidal functions to describe the energy barriers for dihedral rotations. Non-bonding interactions are typically comprised of the Coulomb potential between (partial) charges and the Lennard Jones potential for describing the London dispersion forces. Numerous sets of parameters classically describing all of these interactions have been developed over time, and are referred to as force fields. Some of the most popular force fields include AMBER [61,62], OPLS/AA [63], CHARMM [64], and GROMOS [65]. To reduce computation times, some forcefields lump groups of atoms together, as is the case for the GROMOS force field where CH_2_ groups for example are described as one constituent. Even more coarse-grained force fields have been developed, as well such as the MARTINI [66] force field that was specifically optimized for simulating polymers. The quality of the classical force field is assessed by comparison between simulated and experimental data. Aside reproducing experimental data, the most common analysis tasks of the MD simulations include the evaluation of radial distribution functions, which provide direct insights into the structural organization of the studied systems, and thus the present interactions, as well as the inspection of various correlations functions, from which time constants of present dynamics can be extracted and compared with experimental values. In the case of systems that engage in hydrogen bonding, these can be directly assessed from MD simulations, which experimentally is very difficult to achieve [67]. The interested reader is referred to several references for more details about MD simulations, such as the application of periodic boundary conditions and the pressure and temperature equilibration procedures [68,69].

In a recent review [47] some of us gave an extensive overview about the state of the art of confinement studies of small molecules, such as confined water, small aromatic molecules, alcohols, or carbonic acids [50,70,71,72,73,74,75,76,77] hosted in these materials, and discussed how the confinement affects thermophysical properties such as freezing and melting points of the guest molecules. While confinement effects on small guest molecules with simple physicochemical properties in mesoporous environments are well investigated, until recently not much was known about the local structures of more complex molecules, such as surfactants, in mesoporous confinement. In continuation with the previous review, the present paper gives an overview of a series of newer studies, where more complex molecules are confined inside these materials. The larger number of functional molecular sites permits a larger number of possible interactions, which enables these molecules to form more complex or richer structures than simple small molecules like benzene or pyridine. Of particular interest here is the competition between surface–guest hydrogen bonds and intermolecular (and possibly also intramolecular) guest–guest hydrogen bonds. This review only summarizes the findings since 2020. For a very extensive overview of older work, the reader is referred to the previous review [47].

The rest of this review is organized as follows: Section 2 gives an introduction into the preparation and surface modification of the mesoporous host materials and the investigated surfactants; Section 3 discusses the physicochemical properties of these guest molecules in their bulk phases and their behavior inside the confinement; and the review concludes with a Summary and Outlook into possible future developments in the field.

## 2. Materials and Methods

### 2.1. Host-Materials

Mesoporous silica materials like MCM-41 or SBA-15 combine large and adjustable (via the preparation) pore sizes, specific volumes, and specific surface areas with high thermal stability, low specific weights, and narrow pore-diameter distributions [78,79,80]. Both types of materials are relatively easy to prepare and to functionalize, following, e.g., the synthesis protocol by Grünberg et al. [81,82] or Grün et al. [83] (for details, see refs. [36,80,84]). Their quality, pore dimensions, and surface parameter can be easily determined by the combination of nitrogen adsorption (BET and BJH), ^29^Si SSNMR spectroscopy, and SAXS. Important to note is that freshly prepared samples contain a substantial amount of surface-bound water molecules [49,85,86], which in general have to be removed for confinement [22] studies employing special drying protocols for the preparation of “water-free” silica samples [87].

### 2.2. Probe Molecules

**Figure 1:**
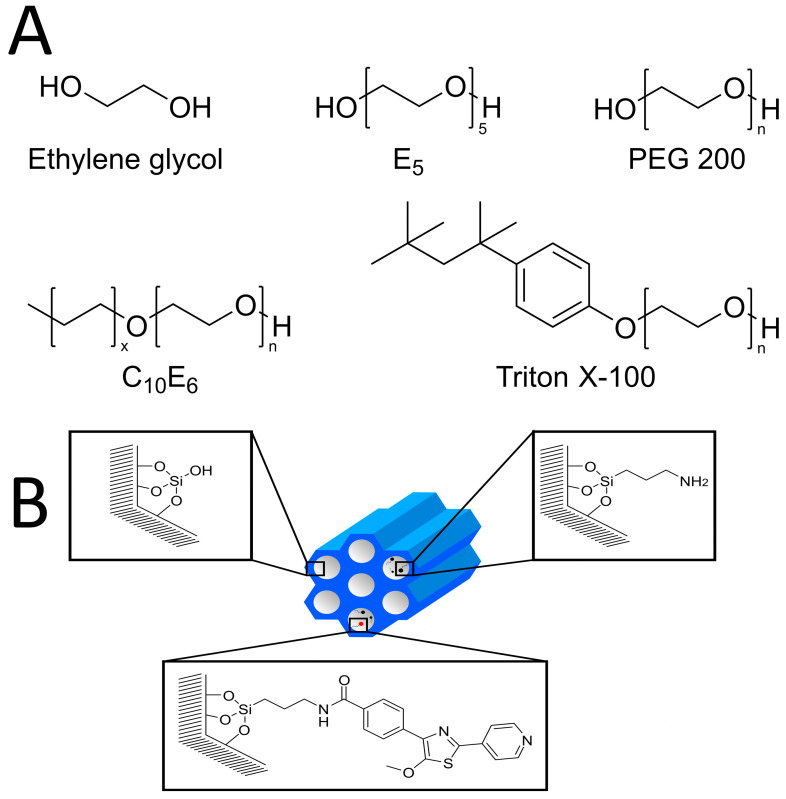
(**A**) Structures of 1-Octanol, EG, and the surfactants studied in this work. Except for E_5_, the surfactants are polydisperse mixtures (exact compositions are given in ref. [88]). (**B**) Sketch of neat and functionalized mesoporous silica material.

The probe molecules considered in this review are water, octanol, ethylene glycol, and the surfactants E_5_, polyethylene glycol, C_10_E_6_, and Triton-X (see Figure 1). Each of these chemical structures contains hydroxy as well as ether moieties, which both can engage in hydrogen-bonding interactions. While octanol, C_10_E_6_, and Triton-X have only a single hydroxyl group, which can interact with the silica surfaces, water, ethylene glycol (EG), and its polymers (PEGs) can interact via two terminal hydroxyl groups. Moreover, in the case of E_5_, C_10_E_6_, and PEG, there is a length-dependent number of ether-oxygens, which can serve as a hydrogen-bond acceptor in competition to the hydroxyl groups.

#### 2.2.1. 1-Octanol

1-octanol, an unbranched saturated fatty alcohol with the molecular formula CH_3_(CH_2_)_7_OH, is commonly employed in the synthesis of esters. Owing to the hydrophilic hydroxy-group and the lipophilic alkyl chain, it is an ideal small model surfactant. It is often employed for evaluating the lipophilicity of pharmaceutical products. A quantitative measure for this is the water octanol partition coefficient or *p*-value K_ow_ [89]. It can be employed, e.g., for estimations of the partitioning of dissolved drug molecules between the cytosol and lipid membranes of living systems in pharmacology, or the behavior of water/oil mixtures in geology or environmental science [90]. Water–octanol mixtures are ideal model systems of the phase behavior of immiscible liquids in bulk and confined phases by combinations of solid-state NMR techniques such as 1D ^1^H-MAS NMR, ^29^Si-CP-MAS (Cross-Polarization Magic Angle Spinning) NMR, and ^1^H/^29^Si-HETCOR-(FSLG)-NMR (Heteronuclear Correlation by Frequency Switched Lee–Goldburg Decoupling) combined with MD simulations are commonly employed in such studies. The combination of these techniques reveals important information such as the distributions of the two liquids inside a confinement, as was shown recently by Kumari et al. in a series of papers [91,92,93].

#### 2.2.2. Ethylene Glycol, Pentaethylene Glycol, and Polyethylene Glycol (PEG)

Ethylene glycol (EG) is the smallest vicinal diol and the simplest example of a polyhydric alcohol. EG finds wide application in the production of polyester fibers and for antifreeze formulations. Owing to the presence of the two hydroxyl groups, it can be employed as a simple model for confined liquids that can interact via several hydrogen bonds with host surface groups. Typical examples of these studies include NMR studies of small confined molecules inside zeolites [22,94,95,96] or mesoporous silica materials [45,97,98,99] and their functionalized derivates [100,101]. The low molecular weight representatives of polyethylene glycols (PEG, H-[O-CH_2_-CH_2_]_n_-OH) possess environmentally benign properties including no toxicity, low vapor pressure, reducing exposure through inhalation and biodegradability. PEG is widely and relatively inexpensively available with an industrial annual production of about 500,000 tons per year [102]. Commercial PEG is manufactured as polydisperse mixtures, where the average molar weight is part of the product name. For example, PEG200 has an average molar weight of approximately 200 g mol^−1^. PEG is a very good solvent for a wide variety of chemicals including some mineral salts [103], which facilitates its use, e.g., in transition metal catalyzed reactions, including heterogeneously catalyzed reactions where the transition metal catalyst is immobilized on a solid material of large surface area [104]. Accordingly, PEG has been increasingly used as a very attractive alternative solvent for Green Chemistry [105]. Its properties as an alternative solvent for chemical synthesis were reviewed in several recent articles [106,107,108]. To further aid these efforts in heterogenous catalysis using PEG as a solvent, they need to be studied under confinement to understand how their physical and chemical properties change under these conditions as a function of the degree of polymerization. Such studies should combine experimental studies employing NMR and thermodynamic measurements with theoretical methodologies such as MD simulation in order to be able to correctly interpret experimental results at the molecular level. In this review, confinement studies of the monomer EG, two different EG polymers, namely E_5_, a monodisperse polymer with chain length of five and a polydisperse polymer with a distribution of chain lengths (PEG200), are reported and compared to the commercial surfactants C_10_E_6_ and Triton X-100 (see Figure 1).

### 2.3. Simulation Methods

As there are a number of excellent reviews on MD simulations [109,110,111], here, only the salient features relevant for this review are shortly summarized. The simulations were carried out using the GROMACS platform [112,113]. For liquids, the typical simulation protocol consisted of the steps of randomly inserting usually 1000 molecules into a virtual box that is chosen in size to be too large, removing close contacts between atoms that may have arisen during the random molecule insertion, letting the density equilibrate at the desired constant simulation temperature and pressure, and finally, after establishing the average density, simulating long enough at constant temperature and volume corresponding to the average density so that the system reaches the diffusive regime, which then allows extraction of the self-diffusion coefficient. For PEG200, simulation times of 300 ns were typical at a temperature of 328 K saving at least 10,000 frames for analysis. The simulations results summarized in this review were mostly obtained with the OPLS/AA force field. As is common, periodic boundary conditions were employed along with a Verlet cutoff scheme [114], and treatment of long-rang electrostatic interactions were employed with a smooth Particle-Mesh Ewald (PME) grid-wise cubic interpolation [115]. Temperature control was established with the Bussi–Donadio–Parinello velocity-rescaling thermostat [116], while pressure was controlled with the Parrinello-Rahman barostat [117,118]. Analysis of the obtained simulations was carried out mainly using modules available with the GROMACS platform augmented by some self-developed script files that can be found along with a very detailed description of the simulation details in Hoffmann et al. [119].

### 2.4. Differential Scanning Calorymetry (DSC)

The DSC analysis were performed using a Heat Flux DSC. In this type of DSC, the sample and the reference are heated through the same heating source. Nitrogen was used as the purge and protective gas during the experiments.

The samples mentioned in this review were prepared and packed under inert conditions and tested within a temperature range of 100 to 300 °C under various heating/cooling rates, depending on the sample. For more detailed information about a specific sample, the reader is referred to the original papers [48,120].

## 3. Exemplary Studies

When studying the temperature-dependent dynamics of confined molecules that readily crystallize, such as water, it is important to consider that confinement usually affects the freezing and melting behaviors. This necessitates the determination whether specific findings relate to the fully liquid state above the melting temperature (*T*_m_) or the partially frozen state below this temperature. In partially frozen states, crystalline regions near the pore center coexist at equilibrium with a liquid layer at the pore wall [45,52,121,122,123]. As specific examples of NMR reorientation and diffusion studies on fully liquid or partially frozen states in nanoscale confinements, we will discuss the dynamical properties of water (H_2_O and D_2_O), ethylene glycol, and LiCl aqueous solutions in native and functionalized mesoporous silica. This approach will provide detailed insights into the dependence of the rotational and diffusive motions of hydrogen-bonded liquids on the size, i.e., the diameter *d*, of the pores and the chemistry of their walls.

### 3.1. Water and Ion Dynamics

**Figure 2:**
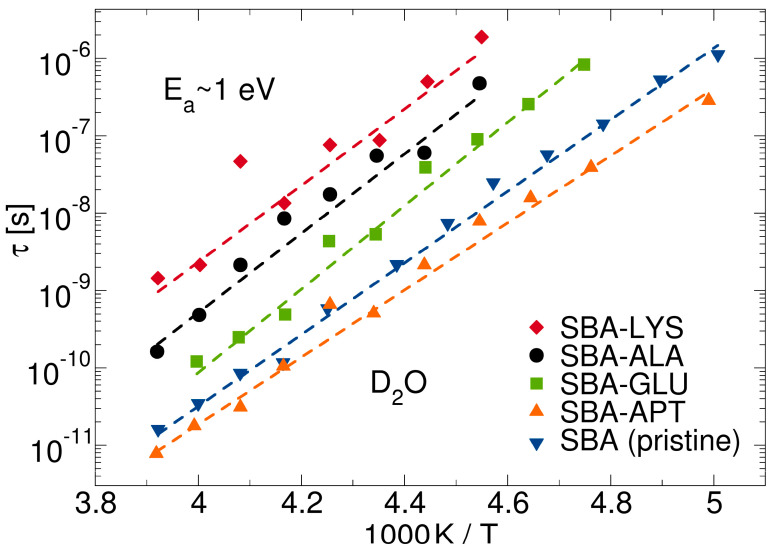
Rotational correlation times *τ* of D_2_O in pristine (*d* = 5.4 nm) [57] and functionalized [124] mesoporous silica. For the functionalization, SBA-15 silica was functionalized via co-condensation with 3-(aminopropyl)triethoxysilane (APTES), yielding SBA-APT (*d* = 6.8 nm). Afterwards, the amino acids lysine (LYS, *d* = 5.8 nm), alanine (ALA, *d* = 5.9 nm), or glutamic acid (GLU, *d* = 5.6 nm) were coupled to this SBA-APT batch. The surface densities of the linked amino acids were 0.4–0.5 nm^−2^. The dashed lines are Arrhenius fits with activation energies of ca. 1 eV.

In partially frozen states, the molecules of the liquid layer interact with the porous framework in their immediate neighborhood very closely, enabling detailed insights into the influence of the confinement chemistry on liquid dynamics. Exploiting this possibility, ^2^H NMR was utilized to investigate D_2_O reorientation near different biomimetic interfaces, specifically near silica walls functionalized with various amino acids [58,124]. It was found that the rotational correlation times *τ* obtained from ^2^H SLR analysis were longer in the functionalized than the pristine silica pores, but with values strongly dependent on the type of the amino acid; see Figure 2. The longest correlation times were observed for lysine (LYS), followed by alanine (ALA) and, finally, glutamic acid (GLU) functionalization. Based on these results, it was concluded that the flexibility of the surface groups is not the decisive parameter for the mobility of neighboring water molecules [58]. Instead, it was proposed that water reorientation is slower near amino acids with basic residues than near those with acidic ones. In addition, it is evident from Figure 2 that, independent of the functionalization, water dynamics exhibited a high temperature dependence in the narrow interfacial layers between the pore walls and the ice cores, described by an activation of about 1 eV, corresponding to nearly 100 kJ/mol.

Other studies employed very narrow confinements with diameters of *d* ≈ 2 nm to fully suppress crystallization of water [53,56,57]. In these cases, a combination of ^2^H SLR and STE studies provided access to the slowdown of water reorientation when approaching a glass transition. An important question of such research was to what degree the dynamics of confined water resembles that of bulk water in the supercooled regime, which is difficult to access due to rapid crystallization. In particular, it was vigorously debated whether or not dynamical crossovers observed for confined water [40,125,126] may be taken as evidence for the existence of a second critical point associated with a liquid–liquid phase transition of bulk water, which was proposed to be at the origin of water’s anomalies [127].

**Figure 3:**
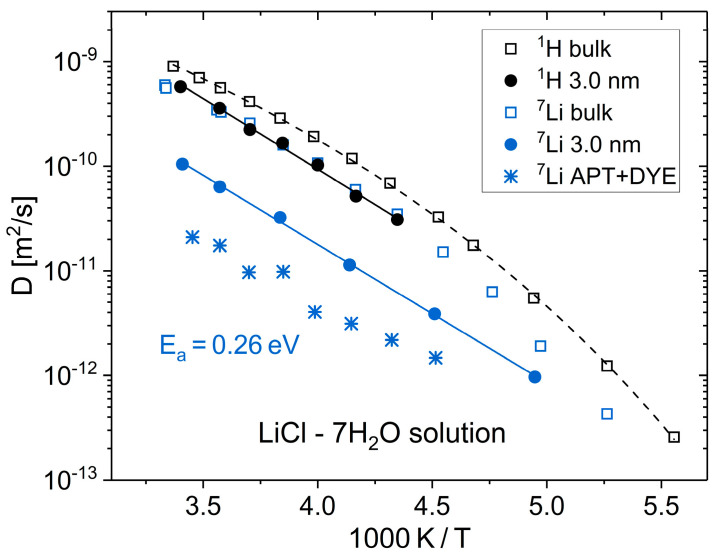
^1^H and ^7^Li diffusion coefficients of a LiCl-7·H_2_O solution in the bulk [128], in pristine silica pores with a diameter of *d* = 3.0 nm [129], and in functionalized silica pores (APT + DYE, *d* = 5.8 nm) [130]. For the functionalization, SBA-15 was functionalized with APTES via co-condensation, and then further modified by adding 4-(5-methoxormatioridin-4-yl)thiazol-4-yl)benzoic acid dye molecules, yielding a grafting density of ~1 dye molecule per nm^2^. The dashed line is a VFT interpolation of the ^1^H diffusion coefficients of the bulk solution. The solid lines are Arrhenius fits of the ^1^H and ^7^Li diffusion coefficients in the pristine pores, yielding the same activation energy of *E*_a_ = 0.26 eV.

Electrolyte solutions in nanoscale confinements are of enormous relevance across various fields. Their applications in heterogeneous catalysis and energy conversion are fundamental to modern society, and ion channels are crucial for the biological functions of living cells. However, many properties of electrolyte solutions in interfaces remain insufficiently understood to this day. The isotope selectivity of NMR was exploited to separately analyze water and ion dynamics for LiCl solutions in the bulk [128] and various confinements [129,130]. Figure 3 shows diffusion coefficients of LiCl–7H_2_O solution obtained from ^1^H and ^7^Li SFG studies, which reflect the mobility of the water molecules and lithium ions, respectively. This composition, which is close to the eutectic one, was chosen to suppress crystallization and enable investigations in a broad temperature range. For the bulk solution, it was found that the ^1^H diffusivity was slightly larger than the ^7^Li diffusivity, and both exhibited a prominent non-Arrhenius temperature dependence typical of many glass-forming liquids. When confining the LiCl–7H_2_O solution to a pristine silica pore with a diameter of *d* = 3.0 nm, the difference of the ^1^H and^7^Li diffusivities increased to nearly an order of magnitude, and the temperature dependence again changed to an Arrhenius behavior with an activation energy of *E*_a_ = 0.26 eV [129]. It was argued that these prominent confinement effects resulted from Stern layer formation at the usually negatively charged silica walls. In a silica material with dye molecules grafted to the inner surfaces, even smaller ^7^Li diffusion coefficients were reported [130]. To rationalize this finding, it was conjectured that these bulky functional groups protrude into the interior of the pores and, in this way, form obstacles for the long-range transport of the highly hydrated lithium ions.

**Figure 4:**
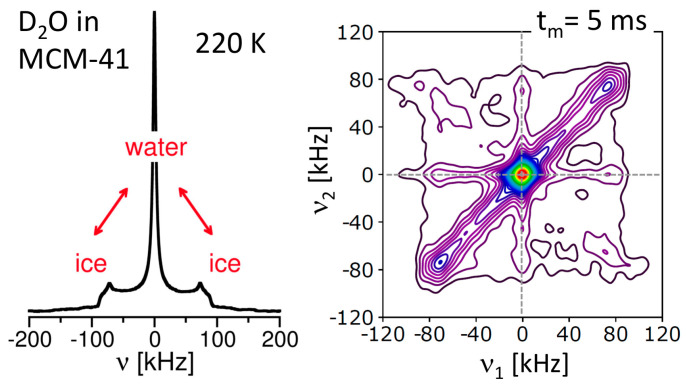
1D and 2D ^2^H NMR spectrum of D_2_O in SBA-15 silica pores with a diameter of *d* = 5.4 nm at 220 K [131]. A mixing time of *t*_m_ = 5 ms was used to record the 2D spectrum. The dashed lines indicate the frequency axes ν_1_ = 0 and ν_2_ = 0 of the 2D spectrum.

Despite their high practical relevance, a comprehensive understanding of the partially frozen states of confined liquids is still lacking. For example, it is unclear whether these two-phase states are ergodic, i.e., whether the crystalline and liquid phases exchange molecules over time. ^2^H NMR spectroscopy yielded important information about the ice–water equilibrium in silica nanopores below the melting temperature *T*_m_ [131]. The method exploited the fact that the 1D ^2^H NMR line shape enabled a discrimination between molecules in the different phases; see Figure 4. Specifically, molecules of the less mobile ice phase contributed a broad Pake pattern (*ν* ≠ 0, in general), while those of the more mobile water phase added a narrow Lorentzian line (*ν* ≈ 0). Under such circumstances, 2D ^2^H NMR spectra provided access to an exchange between both fractions. Specifically, molecules that belonged to water prior to the mixing time (*t*_m_) exhibited *ν*_1_ ≈ 0 and became ice during this period of the 2D experiment so that they showed *ν*_2_ ≠ 0, produced a Pake spectrum along the frequency axis *ν*_1_ = 0. Vice versa, molecules that were a part of the ice phase before the mixing time *t*_m_ and of the water phase afterwards contributed such line shape along *ν*_2_ = 0. Together, a cross-like 2D spectral intensity along the frequency axes indicated ice–water exchange during the mixing time *t*_m_. In a 2D ^2^H NMR spectrum of D_2_O in SBA-15 pores measured at 220 K for a mixing time of *t*_m_ = 5 ms, a cross-like intensity along the frequency axes was clearly observed; [131] see Figure 4. Unlike in a ^1^H NMR approach [132], where spin diffusion was faster and at the underlying process of an exchange between the magnetizations of ice and water phases, the ^2^H NMR findings could be traced back to an exchange of molecules between both phases. Explicitly, a detailed analysis of the mixing-time dependence of the cross-like and other 2D spectral intensities revealed that the residence time of a molecule in either phase was characterized by an exchange time of 5.7 ms at 220 K. Thus, the ice–water equilibrium was highly dynamic, or, in other words, ergodicity restoration occurred relatively fast for the two-phase state of water in nanoscale confinement. In this context, it should be mentioned that the crystal structure of confined ice was discussed for years, and the existence of stack-disordered ice comprising interlaced layers of cubic ice (I_c_) and hexagonal (I_h_) ice was proposed in recent studies [133,134].

### 3.2. Octanol

In the following, it is described how solid-state NMR techniques combined with MD simulations and thermodynamic techniques can be employed to investigate the structural arrangement and dynamics of confined molecules, employing monohydric alcohol 1-octanol [92,135] as a model compound for surfactants. The techniques employed for these investigations were originally developed for the study of confined isobutyric acid [136,137].

**Figure 5:**
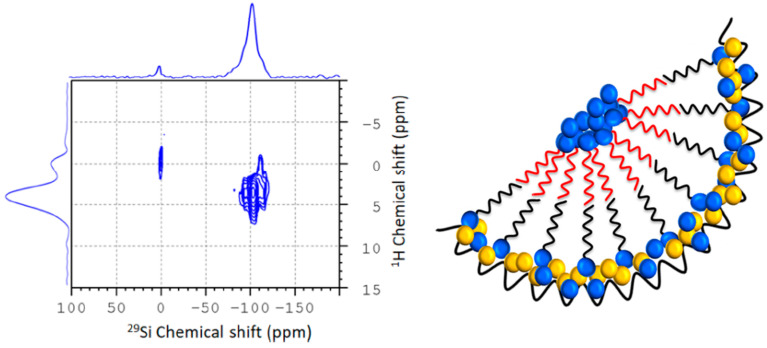
**Left**: Low temperature ^1^H/^29^Si CP-MAS FSLG HETCOR obtained with 9 ms contact time of an 80:20 mol% 1-octanol:water mixture confined in SBA-15. Referencing of the ^1^H-dimension was performed by employing the technique described in ref. [93]. **Right**: Graphical visualization of a feasible bilayer formation of 1-octanol (blue) inside the pore. Water molecules (blue) are concentrated near the pore wall, as well as in the pore center (adapted from Kumari et al. [92]).

The structural arrangement of the octanol molecules [92] can be investigated with the application of ^1^H/^13^C CP-MAS FSLG HETCOR experiments (see Figure 5, left panel). These HETCOR experiments are sensitive to the magnetic dipolar interaction, thus providing information about the distance between the carbon nuclei of the octanol and the carbon and silica protons of the octanol and the silica host, respectively. By variation of the contact time in these experiments, it is possible to sense how close various moieties of the confined solvent molecules are to the pore surface. The analysis of the resulting spectra allows to deduce neighborship relations, which can be interpreted in terms of orientations and arrangements of confined molecules relative to the pore surfaces of the silica host (Figure 5, right panel).

**Figure 6:**
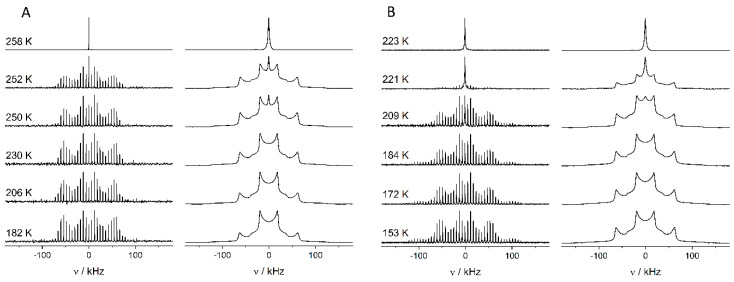

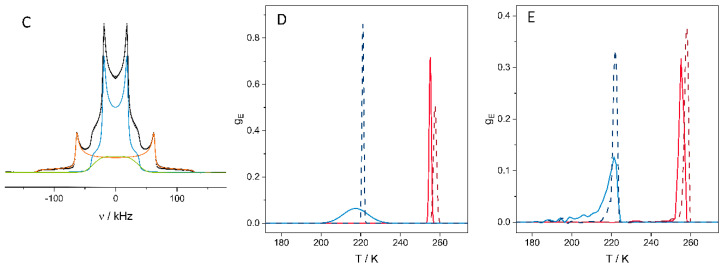
Comparison of ^2^H MAS NMR spectra and solid echo ^2^H spectra of (**A**) bulk octanol-*d*_17_ and (**B**) octanol-*d*_17_ confined in mesoporous SBA-15 as a function of temperature. Exemplary line-shape analysis of a static spectrum and calculated distributions of activation energies are shown in the bottom panels: (**C**) line-shape analysis of the solid echo ^2^H NMR spectrum of bulk octanol-*d*_17_ at 150 K (black) revealing (blue) narrow Pake pattern of methyl deuterons; (orange) broad Pake pattern of methylene deuterons and unstructured component (green) of deuterons with short relaxation times. (**D**,**E**): Distribution of activation energies for the melting process, calculated with the Roessler model (blue: octanol-*d*_17_ in SBA-15, red: bulk octanol-*d*_17_, solid lines: MAS conditions, dashed lines: static conditions). (Adapted from Döller et al. [135]).

The dynamics of 1-octanol-d_17_ in its bulk phase and confined in mesoporous silica SBA-15 were also investigated by a combination of DSC experiments and ^2^H-variable temperature solid-state NMR (solid-echo and MAS NMR experiments) in the region of the solid–liquid phase transition [135]. Compared to previous studies of smaller molecules such as benzene [50,138], naphtalene [139], or bipyridine [140], where relatively broad activation energy distributions were observed, the DSC results could be modeled by the Kissinger model, employing a single activation energy of (313.6 ± 2.1) kJ mol^−1^ for the bulk and a single activation energy of (172 ± 17) kJ mol^−1^ for the confined octanol [141]. The smaller activation energy of the confined octanol is reflected in its lower melting point, which is approximately 38 K below the bulk value (e.g., 219.7 K versus 257.3 K at a heating rate of 5 K min^−1^). The larger uncertainty in the value of the confined molecules is already an indication of larger structural disorder and the coexistence of different species with different melting points [142]. In order to gain further insights into the effects of the confinement on the octanol, the ^2^H-solid state NMR spectra of bulk and confined octanol are compared in Figure 6A,B. Generally, the melting points observed in the NMR experiments agree with those determined using the DSC measurements. Their line-shape analysis (Figure 6C) reveals a superposition of different spectral components. The static spectra below the melting point display a superposition of two Pake patterns with different width and intensity. The broader Pake pattern shows *C*_Q_ ≈ 170 kHz just until the melting point, a value typical for an immobile deuteron of a -CD bond [143]. The quadrupolar coupling constant of *C*_Q_ ≈ 55 kHz of the narrower Pake pattern is characteristic for a CD_3_-group moving around its C_3_-axis in a three-fold jump [144]. Accordingly, the two Pake patterns are assigned to the methyl and methylene deuterons of the alkyl chain. An additional narrow Lorentzian signal appears close to the melting point. This type of signal is characteristic for the onset of melting and the presence of mobile molecules. Below temperatures of 195 K for bulk octanol-*d*_17_ and 170 K for octanol-*d*_17_ in SBA-15, an additional broad and unstructured component is present in the static spectra. The latter is attributed to deuterons whose motions are falling into the intermediate exchange regime and have relatively short effective T_2_ values [145,146]. This interpretation is corroborated with the ^2^H MAS NMR spectra, where only the large and the small Pake pattern are visible, plus the narrow Lorentzian signal close to the melting point of the respective compound, but not the broad unstructured component, due to its short T_2_. The distribution of activation energies for the melting process is calculated from the mole-fractions of the spectral components employing the Roessler model [147] (see ref. [135] for details).

The resulting distributions of activation energies of melting corroborated the results of the Kissinger analysis. For the bulk octanol-*d*_17_, a narrow distribution of a well-ordered crystalline solid was observed [135], and for the confined octanol a broad distribution denoting a melting process involving species in a distribution of different environments and activation energies, and possibly a distribution of rigidly and less-rigidly ordered molecules [71,142], was observed. Moreover, the melting curves for the confined octanol-*d*_17_ exhibit clear deviations between the experimental and calculated curves towards lower temperature, which are indicative for a non-Gaussian distribution of activation energies. Additionally, this non-Gaussian distribution is illustrated by the numerical derivative of the experimental data (Figure 6, right), especially for the confined octanol-*d*_17_ under MAS conditions. For this sample, a shoulder on the low-temperature flank of the main curve is visible, indicating lower melting points for the molecules involved in the pre-melting process compared to the full melting.

In studies of guests that are not fully isotope labelled, it is often necessary to employ DNP enhancement for the characterization of the confined guest molecules. The efficacy of these DNP enhancements in hyperpolarized NMR crucially depends on a good compatibility between the polarizing agent (PA), which is typically a dissolved organic mono- or bi-radical, and the employed frozen solvents, often called the DNP matrix. A homogeneous distribution of the radicals, both in space and orientation, is necessary to achieve good enhancements. In order to investigate this, Döller et al. [148] studied the behavior of four different commercially available DNP polarizing agents confined in the non-ionic model surfactant 1-octanol as analyte and established a novel relative quantification method for the comparison of the proportion of the direct and indirect polarization transfer pathway efficacies, which is able to take concentration effects into account. This study revealed that the hydrophilicity of the PA is the key factor in the way polarization is transferred from the polarizing agent to the analyte.

### 3.3. Ethylene Glycol

**Figure 7:**
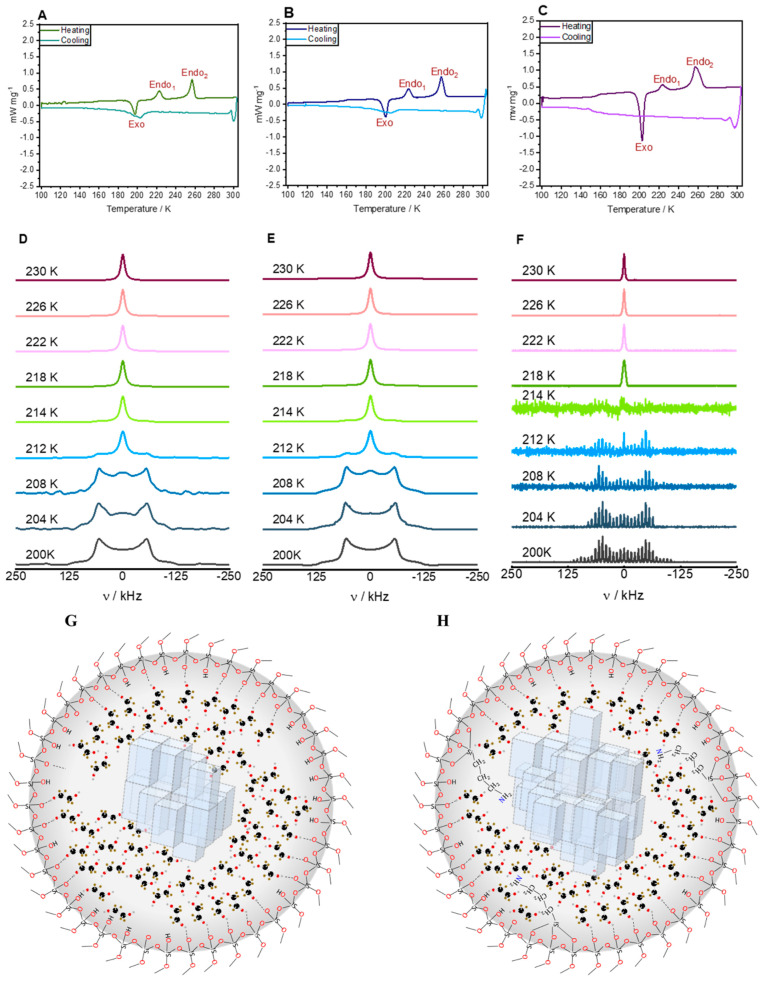
(**A**–**C**): DSC scans of EG confined in SBA-15 decorated with APTES at scan rates of 5 K min^−1^ (**A**), 10 K min^−1^ (**B**), and 15 K min^−1^ (**C**), respectively. At 5 and 10 K min^−1^, only partial freezing is observed during cooling, as evidenced by the broad negative peak near 205 K. As the sample relaxes during heating, the release of heat near 200 K indicates the formation of a crystalline solid that then melts again in two steps near 200 K and 260 K, where the latter is presumed to indicate the presence of EG not confined in the pores. At 15 K min^−1^ (and higher rates, not shown) the small step between 150–160 K during cooling indicates formation of a glass. (**D**–**F**): ^2^H ssNMR spectra obtained in the temperature range between 200 and 230 K for EG-d4 in SBA-15 (sample 2). (**D**) ^2^H static NMR experimental data, (**E**) fitted ^2^H static NMR spectra, and (**F**) ^2^H MAS experimental data. (**G**,**H**): Schematic illustration of the arrangement of EG-d_4_ molecules inside the pores of (**G**) pristine SBA-15 and (**H**) APTES-functionalized SBA-15. This picture includes the formation of crystal-like structures of EG-d_4_ in the middle of the pores, as well as amorphous structures of EG-d_4_ close to the pore wall.

As the starting point of the investigation of larger confined guest molecules who are capable to perform several hydrogen bonding interactions, a study involving partially deuterated ethylene glycol monomer (EG-d_4_) in its bulk phase, confined in SBA-15, as well as APTES-modified SBA-15 was conducted. A combination of thermodynamic measurements, solid-state NMR, and MD simulations were employed [120]. The phase behavior (i.e., melting, crystallization, glass formation, etc.) of EG-d_4_ in these three systems was studied using DSC (Figure 7). Through line-shape analysis of the ^2^H ssNMR spectra recorded at different temperatures, two signal patterns were identified for each of the three investigated systems: a Lorentzian pattern, indicative of a liquid-like state, and a Pake pattern characteristic of a solid-like state. Using a two-phase model, the distribution of activation energies for the dynamic processes in each system was calculated. The spectra reveal an interesting behavior of the confined EG. On the one hand, similar to the previously studied confined 1-octanol [135], the ^2^H NMR spectra indicated the formation of a crystalline solid inside the pores at reduced phase transition temperatures compared to unconfined EG. On the other hand, DSC scans of the same samples, shown exemplarily in Figure 7, indicated the formation of an amorphous glass under rapid cooling. Interestingly, during the heating phase of the DSC temperature cycle, the formed amorphous glass relaxes to form a crystalline solid that later melts at further elevated temperatures. Moreover, the behavior of the EG depends strongly on the surface modification of the SBA-15 host. On non-functionalized surfaces, strong hydrogen-bonding interactions between EG and surface silanol groups were revealed by causing a slowing down of EG dynamics [149].

In contrast, in the case of APTES-functionalized surfaces, where the polar surface-silanol groups are, to a large extent, removed by the binding of the APTES, the surface is far less polar and has a much lower capacity to form hydrogen bonds with EG-molecules [150]. This results in weaker interactions between pore-surface and EG molecules, placing more importance to the EG–EG interactions and a higher tendency to form crystalline EG phases. As a result, a substantially larger portion of EG in the pore remains solid after the first melting event. These effects are schematically shown in the lower panel of Figure 7 (see ref. Haro et al. [120] for more details).

**Figure 8:**
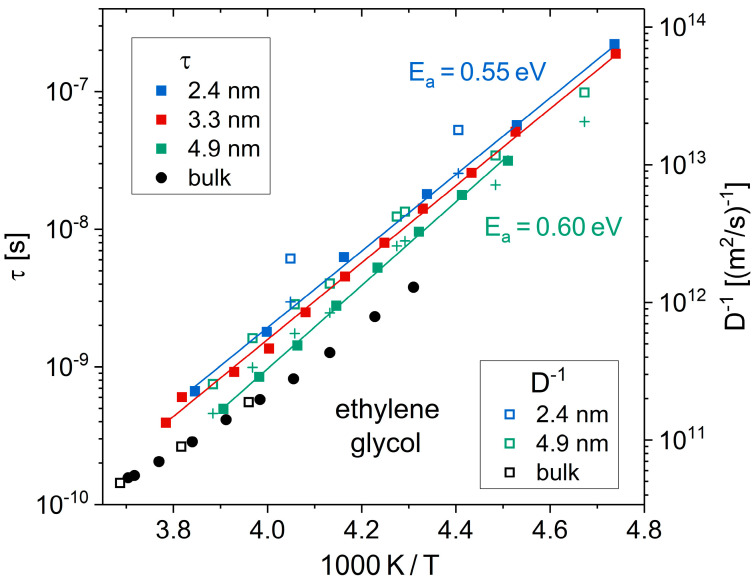
Temperature-dependent correlation times *τ* (solid symbols) and diffusion coefficients *D* (open symbols) of EG in the bulk liquid and in silica pores with the indicated diameters *d* [149]. The axis scaling superimposes *τ* (from ^2^H SLR on EG-d_4_) and *D*^−1^ (from ^1^H SFG on EG-h_6_) data given the SED relation is valid, and the hydrodynamic radius amounts to *R*_H_ = 1.15 Å. The solid lines are Arrhenius fits of the correlation times, yielding activation energies of *E*_a_ = 0.55 eV for *d* = 2.4 nm and *d* = 3.3 nm and *E*_a_ = 0.60 eV for *d* = 4.9 nm. The crosses are correlation times *τ* calculated from the diffusion coefficients *D* using a hydrodynamic radius of *R*_H_ = 0.8 Å and *R*_H_ = 0.9 Å for the pore diameters of *d* = 2.4 nm and *d* = 4.9 nm, respectively.

The rotational motion and translational diffusion of EG in mesoporous silica is studied in detail in refs. [120,149]. To determine the pore-size dependence of these dynamics unaffected by crystallization, a ^1^H and ^2^H NMR study focused on the fully liquid state above *T*_m_ [149]. Correlation times (*τ*) from ^2^H SLR measurements indicated that the molecular reorientation mildly slows down as the pore size decreases to *d* = 2.4 nm; see Figure 8. A slightly more pronounced pore-size dependence was observed for the diffusion coefficients (*D*) from ^1^H SFG experiments. However, both reorientation and diffusion exhibited similar temperature dependencies. In both cases, the non-Arrhenius temperature behavior of the bulk liquid turned into Arrhenius behavior in sufficiently severe confinements. In more detail, the Stokes–Einstein–Debye (SED) relation,

$$D\tau =\frac{2}{9}R_{H}^{2}$$

was obeyed not only in the bulk liquid, where the experimental values of *τ* and *D* indicated a hydrodynamic radius of *R*_H_ = 1.15 Å, but also in the silica pores. However, the different pore-size dependencies of the rotational and translation motions manifested themselves in reduced hydrodynamic radii, e.g., *R*_H_ = 0.8 Å for the narrowest confinement with *d* = 2.4 nm. Correlation times *τ* of EG in lysozyme and elastin matrices obtained from ^2^H NMR SLR and STE studies resembled those in silica pores [151]. In particular, an Arrhenius temperature dependence with an activation energy of *E*_a_ ≈ 0.6 eV was also observed. Thus, the dynamics of EG does not depend on the exact chemistry of a confining framework, at least as long as the latter allows for a formation of hydrogen bonds.

### 3.4. Polyethylene Glycol and Related Surfactants

#### 3.4.1. Experimental Studies

**Figure 9:**
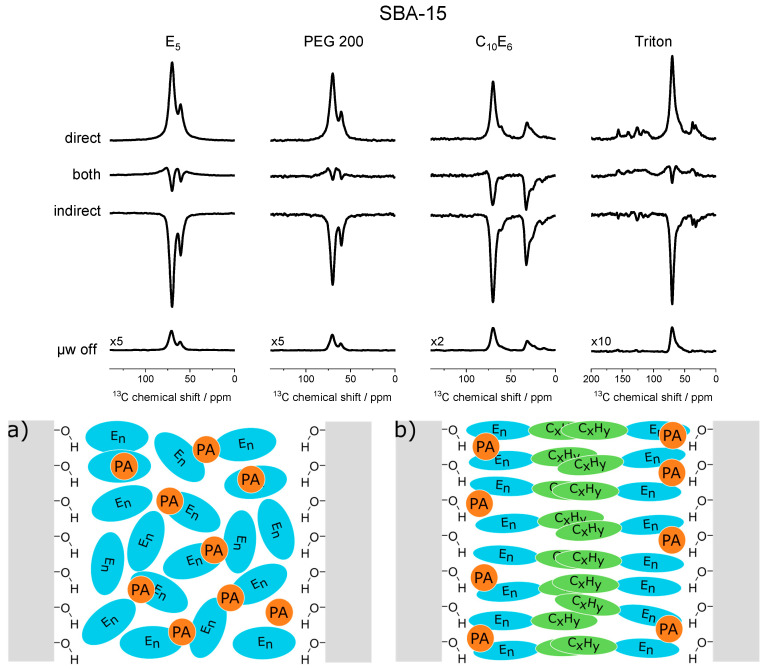
**Upper panel**: DNP-enhanced ^13^C MAS ssNMR spectra revealing the competition between direct and indirect polarization of the confined surfactants. **Lower panel**: Schematic illustration of the distribution of the PA and the surfactant in the pores: (**a**) the hydrophilic surfactants E_5_ and PEG200, and (**b**) the amphiphilic surfactants C_10_E_6_ and Triton. Adapted from ref. [152].

In the next step, the EG polymers E5 and PEG200 were studied in confinement and compared to the commercially available surfactants C_10_E_6_ and Triton X-100 [152]. Two different mesoporous silica materials (SBA-15 and MCM 41) were impregnated with these surfactants. DSC was employed to confirm the confinement of the surfactants in the pores of their host materials. DNP enhanced solid state ^13^C MAS-NMR spectra were recorded for these materials, showing that both the direct as well as the indirect polarization transfer pathways are active for the carbons of the polyethylene glycol moieties of the surfactants (see Figure 9). The presence of the indirect polarization pathway implies the presence of molecular motion with correlation times faster than the inverse Larmor frequency of the observed signals. The intensities of the signals were determined, and an approach based on relative intensities was employed to ensure comparability throughout the samples. From these data, the interactions of the surfactants with the pore walls could be determined. Additionally, a model describing the surfactants’ arrangement in the pores was developed. It was concluded that all carbons of the hydrophilic surfactants, E_5_ and PEG200, interact with the silica walls in a similar fashion, leading to similar polarization transfer pathway patterns for all observed signals. For the amphiphilic surfactants C_10_E_6_ and Triton X-100, the terminal hydroxyl group mediates the majority of the interactions with the pore walls and the polarizing agent. From these data, models of the distribution of the surfactants and PA in confinement can be built (see Figure 9).

**Figure 10:**
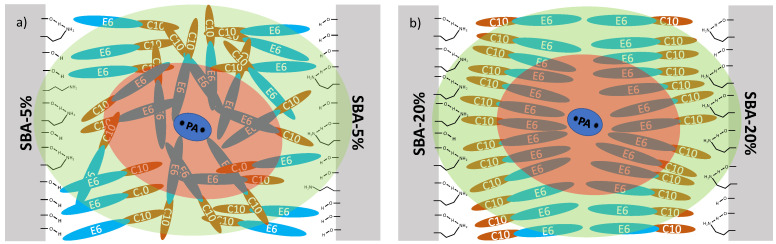
Illustration of (**a**) SBA-5%, (**b**) SBA-20% impregnated with a hydrophilic polarizing agent (PA) in C_10_E_6_. The larger APTES coverage in SBA-20% renders the pore surface to be less polar than in SBA-5%, which supports formation of a structured bilayer arrangement of C_10_E_6_ within the pore. The gray blocks represent the silica pore wall, and the red oval area represents the region around the polarizing agent where nuclei cannot be detected using NMR. The green oval represents the region to which nuclei may receive polarization directly from the polarizing agent. Adapted from Hoffmann et al. [119].

Finally, in ref. [153] it is shown how linewidth and DNP intensities are able to provide very detailed structural information about confined surfactants, employing C_10_E_6_ confined in SBA-15 modified with aminopropyltriethoxysilane (APTES) as an example. The structural interpretations were largely deduced from the trends in linewidths and intensities of the observed dynamic DNP-enhanced solid state ^13^C spectral features originating from the direct and indirect polarization transfer. These findings illustrate that DNP may not only be used to boost signal intensity, but also as a tool to obtain chemical information related to structure and intermolecular interactions. Specifically, for the case of C_10_E_6_, as shown in Figure 10, it was found that at low surface coverage of SBA-15 with APTES (“SBA-5%” where 5: 95 equivalents of APTES: tetraethyl orthosilicate (TEOS) were used during synthesis) the polar portion of the C_10_E_6_ molecules interact competitively with the polar polarizing agent as well as the abundantly present hydroxy surface functional groups. These competitive interactions result in a more disordered assembly of C_10_E_6_ molecules around the polarizing agent, as shown in Figure 10a, compared to the situation shown in Figure 10b where the APTES surface coverage is increased by about 4-fold (“SBA-20%”, i.e., 20:80 equivalents of APTES:TEOS), and thus is overall much less polar. In another set of experiments, we tried to repeat a similar study with 1-octanol as the confined molecule. Unfortunately, the DNP enhancements were too small for this purpose. However, we discovered that the ratio of direct and indirect spectral intensities along with their build-up times of bulk unconfined 1-octanol, can be used as a polarity measure of the polarization agent [148].

#### 3.4.2. Molecular Dynamics Simulations of PEG

The force fields used in MD simulations to describe the bonded and nonbonded interactions between all involved atoms of PEG need to be validated against experimental results. This initial task was recently completed for PEG200, which, to the best of our knowledge, was the first report on MD simulations of mixtures of ethylene glycol oligomers [154]. Widely available forcefields were tested for their accuracy. While some force fields reproduced reasonably well experimental densities and self-diffusion coefficients for diethylene glycol, the agreement of the self-diffusion coefficients and viscosities progressively worsened with increasing oligomer length.

**Figure 11:**
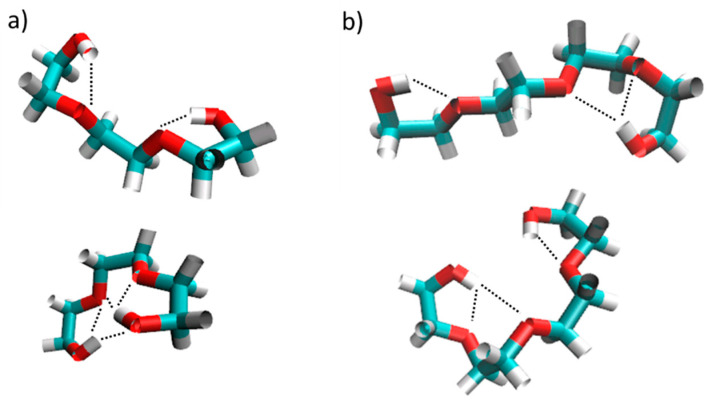
MD simulation sample snapshots of triethylene glycol (**a**) and tetraethylene glycol; (**b**) oligomers illustrating typical structural configurations. The dotted line highlights close proximities of hydroxyl hydrogen atoms to oxygen atoms.

Adjustments to the All-atom optimized potentials for liquid simulation (OPLS-AA) force field regarding the hydroxy end-group charges and the dihedral potential barrier for rotation around the terminal C-C axis were found to improve agreement with experimental data. These adjustments had to be optimized for each oligomer. It was observed that the changes to the OPLS-AA force field reduced overall hydrogen bonding interactions and shifted them from inter- to intra-molecular hydrogen bonding. A remarkable propensity for intramolecular hydrogen bonding was observed for tri- and tetra-ethylene glycol. Typical structural configurations are shown in Figure 11, and illustrate how these two molecules maximize the proximity of the hydroxy hydrogen atoms to intramolecular oxygen atoms. The increased tendency of intramolecular interactions leads to smaller end-to-end distances and radii of gyration, explaining the observed decrease in viscosity and concurrent increase in self-diffusion. Overall, neither the modified nor the unmodified forcefield showed any indication of preferential association of oligomers to form clusters. Instead, PEG200 may be regarded as a random mixture of its ethylene glycol oligomer components. Experimental values of density, viscosity, and self-diffusion coefficient of PEG200, as well as its ethylene glycol oligomer components up to nonaethylene glycol, to which the simulation results were compared to, have only been recently become available, covering a range of temperatures from room temperature to 358 K [155,156]. The trends of these data are important for promoting PEG200 as a solvent, and warrant a brief summary here.

Since water can readily be absorbed from the atmosphere, it is the main impurity of PEG and its oligomer components. The effect of water impurities on the physical properties was tested, and was found to hardly affect these three physical properties up to mole fractions of about 0.15. The temperature dependence showed linearity for density and followed an Arrhenius-type behavior for viscosity and self-diffusion,

(1) $$ln(X\left(T\right))=lnA\;\pm \frac{E_{a}}{RT}$$

where *X*(*T*) represents the temperature-dependent property, and the sign before the second term of the right-hand equation is positive for viscosity and negative for the self-diffusion coefficients, *T* is the temperature, *R* the gas constant, *E_a_* is the activation energy, and *A* is the pre-exponential factor. Interestingly, the *E_a_* values were within experimental uncertainty the same for viscosity and self-diffusion, indicating that the same activation barriers exist for translational motion and momentum transfer. Moreover, with respect to neat oligomer components, the straightforward relationships of the physical properties, with respect to the number of ethylene oxide repeat units, *n*, shown in Equations (2)–(4), could be established allowing the prediction of these properties for higher oligomers.

(2) $$Molar\;Volume,\bar{V}:\bar{V}\left(T\right)/{cm}^{3}\cdot mol=\left(0.0303\;n+0.0079\right)T+30.11\;n+14.38\;$$

(3) $$Viscosity,\eta :\ln\left(\eta (T)/mP\cdot s\right)=-8.619+\frac{456.1n+28851}{RT}$$

(4) $$Self\text{-}diffusion\;coefficient,D:\ln\left(D(T)/m^{2}\cdot s^{-1}\right)=11.268+\frac{456.1n+28851}{RT}$$

**Figure 12:**
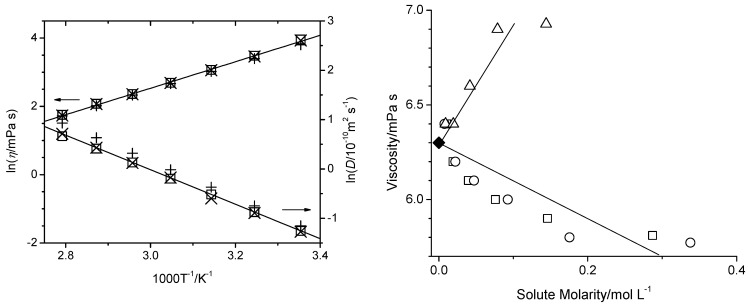
**Left**: Natural logarithms of viscosity (left y-scale) and self-diffusion coefficients (right y-scale) as a function of inverse temperature: experimentally for PEG200 (squares) with least square fit (solid line), experimentally for binary mixture of tri- and hexaethylene glycol with average molar weight of 200 g·mol^−1^ (crosses), and calculated for PEG200 from mole-fraction weighted averages of the individual component properties obtained from Equations (3) and (4) (plusses). **Right**: Bulk viscosity as a function of solute molarity in PEG200 at 358 K for added TEMPO (from BP Bio as squares and AA Blocks as circles) and 5-TBIPA (triangles). The viscosity of neat PEG200 (solid diamond) is taken from Hoffmann et al. [156], and the solid lines serve as guides for the eye.

It was also tested if the composition for PEG200 varies significantly from vendor to vendor, which was not the case. Even if it did, it would be inconsequential because of the remarkable finding that the properties of PEG do not depend on its composition as long as the average molar weight is not changed. This conclusion was based on the observation that a binary tri- and hexaethylene glycol mixture with an average molar weight of 200 g mol^−1^ possesses the same properties as PEG200, as exemplarily shown in Figure 12 for the viscosity and the self-diffusion coefficient. Finally, the binary system of tri- and hexaethylene glycol showed ideal mixing behavior. Thus, the three investigated physical properties were found to be reasonably predictable for PEG200 by mole-fraction weighted averaging of the properties of their individual ethylene glycol oligomer components, as can be seen in the left panel of Figure 12.

Overall, these findings illustrate that applications using PEG200 as solvent should be robust with respect to tolerance to batch-to-batch variations from the same or different vendor sources and levels of water impurity. In preparation for future MD simulations of solutions with PEG as the solvent, experimental data have also been measured for two different solutes of interest in PEG200, namely 2,2,6,6-tetramethylpiperidinyloxyl (TEMPO) and 5-tert-butylisophthalic acid (5-TBIPA) [157]. 5-TBIPA was chosen as a model reagent for the synthesis of MOFs [158]. TEMPO is a widely used stable free radical with applications in chemical transformations [159,160], particularly as a redox catalyst [161]. It was primarily chosen in that study because many polarization agents in DNP-enhanced NMR studies are based on TEMPO as the free radical moiety [162,163,164,165,166,167,168]. As exemplified for the viscosity (right panel of Figure 12), the addition of 5-TBIPA increases solution density and viscosity. Combined with the finding that 5-TBIPA consistently self-diffuses at about half the rate as PEG200 at all investigated experimental conditions [157], this suggests strong attractive solute–solvent interactions, likely through hydrogen bonding interactions. In contrast, the addition of TEMPO causes lower solution densities and viscosities, suggesting that the solute–solvent interactions of TEMPO lead to an overall weakening of the intermolecular interactions present compared to neat PEG200. Furthermore, while the viscosity increases with 5-TBIPA addition, it was found that the PEG self-diffusion remains essentially unaffected, contradicting the Stokes–Einstein relation

(5) $$D=\frac{k_{B}T}{\xi \pi \eta r},$$

where viscosity and self-diffusion are inversely proportional to each other at constant temperature (*k_B_*: Boltzmann constant, *T*: temperature, *r*: hydrodynamic solute radius, *ξ*: a constant typically between values of 4–6).

**Figure 13:**
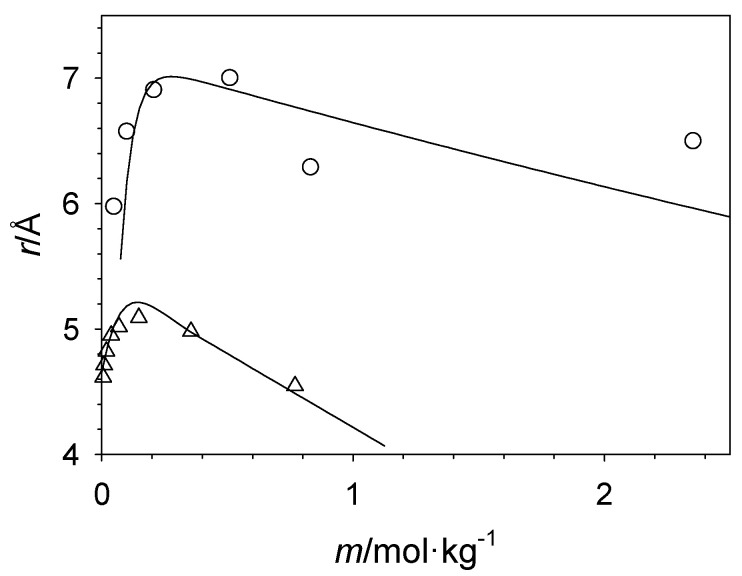
Apparent average solute radii as a function of solute concentration for C_10_E_6_ in cyclohexane (circles) at 283.15 K and for 1-ethyl-3-methylimidazolium bis(trifluoromethylsulfonyl)amide in dichloromethane at 288.15 K (triangles). The lines are guides for the eye.

Deviations from the Stokes–Einstein relation were also reported for the PEG-related surfactant C_10_E_6_ dissolved in cyclohexane [169,170]. In contrast to PEG, the additional alkyl chain leads to the formation of reverse micellar aggregates. The size of the aggregates increases with increasing surfactant concentration. Addition of water further increases the size of the aggregates, as water preferentially resides inside the reverse micelle due to its interactions with the hydrophilic heads of the surfactant compared to the hydrophobic cyclohexane continuous phase. Thus, the water is in a prison consisting, in this case, of reverse micelles in a nonpolar fluid medium. Using the independently measured surfactant self-diffusion coefficients and the solution viscosity, the calculated radii using the Stokes–Einstein relation undergo a maximum as a function of surfactant concentration instead of continuously increasing. This can be explained by a change in mass transport where at low solute concentrations all surfactant solute species (monomers, dimers, small aggregates) contribute to the average self-diffusion coefficient, while at a high-solute concentration, the aggregates have become too large to significantly contribute to the mass transport that is thus now dominated by the monomer species (along with dimers/lower oligomers). Pictorially, at a high-solute concentration, the surfactant monomers are hopping from aggregate to aggregate, which in comparison are stationary. This also implies that there remain significant numbers of surfactant monomers present even at high surfactant concentrations, which shows that reverse micellar formation proceeds through a series of chemical equilibria steps, monomer to dimer, dimer to trimer, etc. Such gradual formation of the reverse micelle is in sharp contrast to the typical micelle formation in aqueous solution where a large number of surfactant solutes form in one step one very large micellar aggregate, and the chemical equilibrium is shifted so far to the micelle formation that the concentration of unaggregated surfactant monomers is negligibly small. The same phenomenon of an apparent maximum of aggregate size with increasing solute concentration has also been observed for some ionic liquids dissolved in solvents of low polarity [71,72,171,172], as shown in Figure 13 exemplary for 1-ethyl-3-methylimidazolium bis(trifluoromethylsulfonyl)amide in dichloromethane, illustrating that this phenomenon is more broadly applicable. Interestingly, more reasonable aggregate radii could be obtained when employing the ratio of surfactant solute and cyclohexane solvent self-diffusion coefficients, i.e., without the viscosities entering the evaluations. This shows how viscosity and self-diffusion are decoupled in these systems thus leading to an apparent breakdown of the Stokes–Einstein relation.

## 4. Summary and Outlook

This paper reviews recent advances in characterizing, in particular, surfactants confined in microporous and mesoporous materials, employing solid-state NMR techniques, calorimetry, and MD simulations. It is shown that the combination of these techniques is capable of revealing detailed insights into the structural arrangement and dynamics of the confined guest molecules, which arise from the interplay between guest–guest and guest–host interactions. It is shown that the simultaneous presence of multiple hydrogen-bonding sites and polar/non-polar moieties in the guests leads to the formation of ordered structures, despite the cramped surroundings inside the pores. A number of examples from the groups of the authors were given to highlight this fascinating emergence of order in the narrow pores. Finally, the review concludes with some thoughts on the future direction of the field. It is anticipated that, due to advancements in MD simulation techniques including the recently reviewed advancements of extracting NMR relevant parameters directly from aiMD simulations [173], it will be more and more feasible to predict the structural arrangements of the confined guest molecules a priori, and later probe these predictions by the combination of calorimetry, solid-state NMR, and DNP.
